# Divergent impacts of glycemic control on mortality and complications in patients with early-versus late-onset type 2 diabetes: A retrospective cohort study

**DOI:** 10.1371/journal.pone.0322886

**Published:** 2025-05-23

**Authors:** Haipeng Ma, Jitao Zhang, Bing Meng, Kai Wang, Yuhong Li, Na Liang

**Affiliations:** 1 Department of Ophthalmology, Handan City Eye Hospital (The Third Hospital of Handan), Handan, Hebei Province, PR China; 2 Department of Laboratory Medicine, The First Hospital of Handan, Handan, Hebei Province, PR China; Instituto Nacional de Cardiologia Ignacio Chavez, MEXICO

## Abstract

**Aims:**

To investigate whether optimal glycemic control is associated with all-cause mortality, cardiovascular disease mortality, diabetes-related mortality, cancer-related mortality, and complications among individuals with early-onset and late-onset T2D.

**Methods:**

We conducted a retrospective cohort study using data from the U.S. National Health and Nutritional Examination Survey (NHANES)1999–2818. Optimal glycemic control was defined as HbA1c<7%, and poor glycemic control as HbA1c≥9%. Mortality and underlying causes of death were ascertained by linkage to national death records through 31 December 2019. Cox proportional hazards regression models adjusted for age, sex, race, education, body mass index (BMI), hypertension, smoking status, alcohol consumption, and physical activity were used to estimate hazard ratios (HRs) and 95% confidence intervals (CIs) for associations between HbA1c levels and mortality. Logistic regression models with the same covariates were employed to calculate odds ratios (ORs) and 95% CIs for complications, supplemented by sensitivity analyses to evaluate the robustness of the findings.

**Results:**

Among the 5946 participants with diabetes, 18.8% were classified as having early-onset T2D (aged < 40 years), 28.7% as having late-onset T2D (aged ≤ 60 years), and 52.5% had average-onset T2D. For individuals with early-onset T2D, the poorly controlled group (HbA1c≥9%) had HRs of 2.00 (95% CI, 1.30–3.09; P = 0.002) for all-cause mortality and 10.04 (95% CI, 2.57–39.32; P = 0.001) for diabetes-related mortality versus the optimal controlled group (HbA1c<7%). The poorly controlled group had odds of 1.80 (95% CI, 1.10–2.94; P = 0.022) for retinopathy and 2.54 (95% CI, 1.65–3.92; P < 0.001) for chronic kidney disease (CKD) versus the optimal controlled group. For individuals with late-onset T2D, the HRs were 0.87 (HR 0.87; 95% CI, 0.54–1.40; P = 0.561) for all-cause mortality and 1.24 (95% CI, 0.33–4.67; P = 0.751) for diabetes-related mortality compared with the optimal controlled group. The poorly controlled group had odds of 2.12 (95% CI, 1.32–3.41; P = 0.002) for retinopathy and 2.30 (95% CI, 1.45–3.63; P = 0.001) for CKD versus the optimal controlled group.

**Conclusion:**

Optimal glycemic control was associated with a reduced risk of all-cause mortality, diabetes-related mortality, retinopathy, and CKD in individuals with early-onset T2D; however, in individuals with late-onset T2D, this correlation was limited to lower risks of retinopathy and CKD. These findings suggest that glycemic control strategies should be tailored on the basis of the age of diabetes onset.

## Introduction

Diabetes mellitus affects approximately 1 in 10.5 adults worldwide, with 90% of cases being T2D. This condition has become a critical public health challenge of the 21st century [1,2]. T2D is a chronic metabolic disorder characterized by progressive insulin resistance and subsequent hyperglycemia [3]. Prolonged hyperglycemia increases the risk of microvascular and macrovascular complications, leading to premature death or disability [4]. Therefore, controlling hyperglycemia is fundamental to diabetes management [5]. Historically, the main strategy used to reduce complications of T2D has been intensive glycemic control [6]. Importantly, T2D often occurs in middle-aged or older adults [7], so the above conclusions are drawn mainly for this patient group. However, an increasing number of young people are also being diagnosed with diabetes, and it remains unclear whether optimal glycemic control has different correlations with mortality and complications in individuals with early-onset and late-onset T2D.

The classification of T2D into early-onset (diagnosis <40 years) and late-onset (diagnosis ≥60 years) disease is not merely a chronological distinction but reflects profound differences in pathophysiology, disease trajectory, and clinical management [8,9]. Compared with late-onset T2D, early-onset T2D is characterized by accelerated β-cell dysfunction and severe insulin resistance [10]. Younger individuals often present a disproportionately high burden of visceral adiposity and metabolic syndrome, driving rapid β-cell exhaustion [10,11]. Critically, the extended disease duration in early-onset T2D, often spanning decades, amplifies cumulative exposure to hyperglycemia, leading to earlier onset of microvascular complications and premature cardiovascular events [12,13]. Early-onset patients demonstrate an attenuated response to conventional stepwise therapy [14]. Notably, since the 2000s, the incidence and prevalence of T2D in young people have increased dramatically [8], emphasizing the importance of researching early-onset T2D. Additionally, the optimal HbA1c management in this population remains poorly defined [15,16].

Therefore, this study aimed to investigate any disparities in the associations of glycemic control with mortality and diabetes-related complications between individuals with early- and late-onset T2D. Moreover, owing to the relatively low prevalence of T2D in young people, data on early-onset T2D are scarce, which impedes the recruitment of such individuals for research studies. To fill these knowledge gaps, we conducted a comprehensive assessment of the associations between HbA1c levels and various outcomes, including all-cause mortality, CVD-related mortality, diabetes-related mortality, and diabetes-related complications. This investigation was carried out among a large, nationally representative sample of U.S. adults with both early-onset and late-onset T2D.

## Materials and methods

### Research design

The NHANES is conducted in the U.S. by the National Center for Health Statistics of the Centers for Disease Control and Prevention. It began in the early 1960s and was conducted as a series of surveys focusing on different population groups or health topics [17]. The survey is unique in that it combines interviews, standardized physical examinations and laboratory tests [18]. The full methodology of data collection is available, and the data used in this study are publicly available (https://wwwn.cdc.gov/nchs/nhanes/default.aspx).

In this retrospective cohort study, we included participants with diabetes (≥18 years of age) from 10 cycles of the NHANES from 1999--2018. We defined diabetes on the basis of participants’ self-reported physician diagnosis of diabetes. In total, 6699 participants met the diagnostic criteria for diabetes. Participants were excluded if they were pregnant (n = 18), as gestational diabetes requires distinct management strategies that may confound the analysis of T2D-specific outcomes. Individuals with type 1 diabetes (n = 17) were excluded because of differences in disease etiology and treatment. Participants with missing HbA1c data (n = 609) were excluded to ensure accurate categorization of glycemic control. A total of 5946 participants with T2D were included. We divided the participants into three groups according to age at diagnosis of T2D (early-onset: < 40 years of age, average-onset: 40 ≤ or < 60 years, and late-onset: > 60 years) [9]. According to the results of epidemiological research and clinical practice, the average age of diabetes diagnosis falls between 40 and 60 years in most cases. Among those included, 1119 participants were in the early-onset cohort, and 1712 participants were in the late-onset cohort.

### Ethical approval declarations

The survey protocols of the NHANES were approved by the Research Ethics Review Board of the National Center for Health Statistics (https://www.cdc.gov/nchs/nhanes/irba98.htm), and all survey participants provided written informed consent. Therefore, no further ethical approval or informed consent was needed.

### Exposure measurement

We categorized HbA1c levels measured by the NHANES team into three groups: optimal controlled (HbA1c level, < 7%) [19], moderately controlled (7%–8.9%) [20], and poorly controlled (≥9%) [20]. HbA1c was measured in the first laboratory on the Primus CLC330 (from 1999–2004) and then in the second laboratory on the Tosoh A1C 2.2 Plus (from 2005–2006). From 2007–2010, HbA1c was measured in the second laboratory on the Tosoh A1C G7 [20]. From 20011–2018, HbA1c was measured in the second laboratory at Tosoh A1C G8.

### Prescription medications

The participants were asked to report prescription medications they had taken in the past 30 days and to bring medication bottles to the examination, where the information was documented. Insulin was determined on the basis of a review of medications provided by the participants or self-reported information, as not all participants may have brought their insulin vials or pens to the examination.

### Ascertainment of mortality

We utilized the NHANES Public-Use Linked Mortality File, which is linked to the National Death Index (NDI) data through 31 December 2019 (https://www.cdc.gov/nchs/data-linkage/mortality-public.htm). To determine the mortality status of our study participants, we used a probabilistic matching algorithm. The NDI is a centralized database [21] of all deaths in the United States. The cause-specific mortality data available in the NDI have been shown to accurately classify deaths. We identified the underlying cause of death via the International Statistical Classification of Diseases, 10th Revision [22].

### Assessment of covariates

The participants’ height (cm) and weight (kg) were measured to determine their BMI. BMI was calculated as weight in kilograms divided by height in meters squared, normal was defined as a BMI < 25 kg/m^2^, overweight was defined as a 25 kg/m^2^ < BMI < 29.9 kg/m^2^, and obesity was defined as a BMI ≥ 30 kg/m^2^ [23]. Hypertension was defined as an SBP value ≥140 mmHg and/or a diastolic blood pressure value ≥90 mmHg [24]. The laboratory methods used to measure HbA1c, the lipid profile, and the levels of ALT, AST, creatinine, blood urea nitrogen (BUN), fasting insulin, and fasting plasma glucose are reported in detail in the NHANES. Insulin resistance was estimated via homeostasis model assessment (HOMA-IR) via the following formula: fasting insulin (μU/mL) * fasting plasma glucose (mmol/L)/22.5 [25]. Chronic kidney disease [26] was identified on the basis of an estimated glomerular filtration rate of less than 60 mL/min/1.73 cm^2^, which was calculated via the Chronic Kidney Disease Epidemiology Collaboration equation [27]. The participants were classified as nonsmokers, former smokers, or current smokers according to their responses about smoking. Educational level was calculated by the highest grade or level of school or the highest degree. The physically active levels were determined via survey questionnaires. Being physically active was defined as participating in moderate-intensity or vigorous sports, fitness programs, or recreational activities for more than 10 minutes per week [28].

### Statistical analysis

All analyses were conducted using R Statistical Software, version 4.2, accounting for the complex survey design of the NHANES. We used appropriate weighting for each analysis, as suggested by the National Center for Health Statistics. Data are expressed as numbers and weighted proportions for categorical variables and as weighted means ± SDs for continuous variables. Variables with a skewed distribution were naturally log-transformed to meet normality criteria. The participants were stratified into 3 groups according to their HbA1c levels. Linear regression for continuous variables and the χ^2^ test for categorical variables were used to detect differences across the 3 groups. Multivariate Cox proportional hazards regression models were used to compute HRs and 95% CIs for the associations of HbA1c levels with the risk of all-cause mortality, CVD-related mortality, diabetes-related mortality and cancer-related mortality. Multivariable logistic regression models were used to compute ORs and 95% CIs for the associations of HbA1c levels with the risk of retinopathy, CKD and CVD. Cox proportional hazards and logistic regression models were adjusted for age, sex, race, education, body mass index, duration of diabetes, hypertension, smoking, drinking, physical activity, diabetes mellitus treatment, comorbidities (retinopathy, CVD, tumor), ALT, AST, BUN, SUA, TG, TC, HDL, LDL, and CRP. Schoenfeld residuals tests and time-dependent covariate analysis were used to confirm that the proportional hazards assumption was met for all covariates in our Cox models. Stratified analyses were performed in the strata of age, sex, race or ethnicity (white and nonwhite), duration of diabetes (<5 years, 5–10 years, ≥ 10 years), BMI (<25, 25–29.9, ≥ 30 kg/m^2^), hypertension (yes or no), smoking (never, ever or now), alcohol intake, drinks/day (0, 1–2, > 2), physical activity (low intensity, moderate intensity, high intensity), hyperlipidemia (yes or no), CKD (yes or no), and diabetes mellitus treatment (insulin, insulin+oral medications, oral medications, no pharmacotherapy). To model potential nonlinear relationships, weighted restricted cubic splines (RCS) with 3 knots at the 10th, 50th, and 90th HbA1C percentiles were used to visualize associations between HbA1c levels and mortality or diabetes complications. In addition, several sensitivity analyses were conducted to examine the robustness of the main results. First, to minimize the possibility of reverse causality and exclude secondary diabetes due to pancreatic pathology (e.g., chronic pancreatitis or pancreatic cancer), we excluded patients with pancreatic diseases and tumors at the baseline examination. Second, we evaluated participants who died within 2 years of follow-up to account for potential survival bias. Third, participants who had a history of anemia at baseline were further excluded to reduce measurement bias in HbA1c interpretation, as anemia may independently affect glycated hemoglobin levels. Fourth, participants who had a history of CVD were excluded to isolate the direct association between HbA1c and outcomes without confounding by pre-existing cardiovascular morbidity. A two-tailed value of P < 0.05 was considered statistically significant.

## Results

### Characteristics of the participants at baseline

A total of 5946 participants were diagnosed during the NHANES 1999–2018, including 18.8% early-onset T2D participants who had a diagnostic age < 40 years, 28.7% late-onset T2D participants who had a diagnostic age ≥ 60 years and 52.5% with average-onset T2D. The characteristics of the study sample are presented in Table 1. The participants had a mean age of 62.0 (±13.2) years and were predominantly male (51.7%). Among participants with early-onset T2D, the mean age at diagnosis was 31.1 (±6.2) years, with a mean disease duration of 17.1 (±13.9) years. In this group, 55.4% were identified as women. Regarding the treatment of diabetes, 41.2% used insulin, 38.7% relied on oral hypoglycemic drugs, and 20.1% did not receive medication. Among the participants with late-onset T2D, the mean age at diagnosis was 66.8 (±6.0) years, the mean disease duration was 6.6 (±5.4) years, and 45.5% were women. In the treatment of diabetes, 16.8% of participants used insulin, 67.4% took oral hypoglycemic drugs, and 15.9% did not receive treatment. In addition, early-onset participants had higher BMI, fasting glucose, HbA1c, insulin resistance index, CRP, total cholesterol, total triglycerides, LDL cholesterol and eGFR. Late-onset participants had higher BUN, creatinine, and SUA levels. Both early- and late-onset participants had more severe insulin resistance and CRP levels in the high HbA1c group (S1 and S2 Tables). Among participants with early-onset T2D, approximately 40% had HbA1c levels below 7%, whereas among participants with late-onset T2D, over 60% had HbA1c levels below 7%. Notably, this trend remained unaltered from 1999–2018 (S1 Fig).

**Table 1:** Basic demographic, behavioral, and biochemical profiles and complication characteristics of patients with diabetes by age at diagnosis in the NHANES (1999--2018).

	Total	Early-onset	Late-onset	*P*
Participants, n	5946	1119	1712	
Characteristic				
Gender, n(%)				<0.001
Male	3072 (51.7)	499 (44.6)	933 (54.5)	
Female	2874 (48.3)	620 (55.4)	779 (45.5)	
Age, years, mean(SD)	62.02(13.2)	48.2 (13.5)	73.5 (6.2)	<0.001
Age at diagnosis, mean (SD)	49.7 (15.4)	31.1 (6.2)	66.9 (6.0)	<0.001
BMI, mean (SD)	32.1 (7.5)	33.8 (8.5)	30.3 (5.9)	<0.001
Weight status, n(%)				<0.001
Normal: BMI of < 25,	832 (14.4)	131 (12.1)	280 (17.0)	
Overweight: BMI of 25 to < 30	1746 (30.3)	259 (23.9)	600 (36.4)	
Obese: BMI of ≥ 30	3181 (55.2)	693 (64.0)	767 (46.6)	
Race, n(%)				<0.001
Mexican American	1222 (20.6)	257 (23.0)	283 (16.5)	
Other Hispanic	533 (9.0)	121 (10.8)	118 (6.9)	
Non-Hispanic White	2115 (35.6)	311 (27.8)	819 (47.8)	
Non-Hispanic Black	1528 (25.7)	318 (28.4)	345 (20.2)	
Other Race	548 (9.2)	112 (10.0)	147 (8.6)	
Eduactional level, n (%)				<0.001
High school or less	3593 (60.4)	633 (56.6)	1103 (64.4)	
Some college	1510 (25.4)	338 (30.2)	373 (21.8)	
College graduate	843 (14.2)	148 (13.2)	236 (13.8)	
Insurance, n(%)				<0.001
Any insurance	5199 (87.7)	882 (78.7)	1645 (96.1)	
Uninsured	747 (12.6)	237 (21.3)	67 (3.9)	
Hypentension, n(%)	4429 (74.5)	718 (64.2)	1374 (80.3)	<0.001
Smoking, n(%)	2050 (34.4)	273 (24.4)	736 (43.0)	<0.001
Alcohol(drinks/day), n(%)				<0.001
0	3308 (55.6)	536 (47.9)	1078 (63.0)	
1- 2	1858 (31.2)	354 (31.6)	535 (31.2)	
>2	780 (13.1)	229 (20.5)	99 (5.8)	
Physical activity, n(%)				<0.001
Low intensity	3213 (54.0)	525 (46.9)	1004 (58.6)	
Moderate-intensity	1834 (30.8)	337 (30.1)	536 (31.3)	
High-intensity	899 (15.1)	257 (23.0)	172 (10.0)	
The duration of diabetes, mean (SD)	12.2 (12.0)	17.1 (13.9)	6.6 (5.4)	<0.001
Diabetes mellitus treatment, n(%)				<0.001
Insulin	781 (13.1)	246 (22.0)	131 (7.7)	
Insulin+ oral medications	849 (14.3)	215 (19.2)	155 (9.1)	
Oral medications	3424 (57.6)	433 (38.7)	1154 (67.4)	
No pharmacotherapy	892 (15.0)	225 (20.1)	272 (15.9)	
Comorbidities, n(%)				
Retinopathy	1321 (22.2)	335 (30.0)	250 (14.6)	<0.001
CVD	1411 (23.7)	209 (18.8)	511 (29.8)	<0.001
Tumor	843 (14.2)	80 (7.1)	399 (23.3)	<0.001
Biochemical profile, mean(SD)				
Glucose (mmol/L)	9.0 (3.9)	10.12 (4.8)	8.04 (2.8)	<0.001
HbA1c(%)	7.5 (1.8)	8.03 (2.2)	6.95 (1.4)	<0.001
HOMA-IR	9.2 (15.8)	11.1 (18.6)	6.9 (10.4)	<0.001
C-reactive protein(mg/dL)	2.6 (7.9)	3.0 (6.8)	1.9 (5.6)	0.001
ALT(U/I)	25.5 (26.8)	28.4 (46.1)	22.6 (12.4)	<0.001
AST(U/I)	25.5 (20.2)	26.0 (25.6)	24.7 (11.6)	0.162
eGFR(ml/min/1.73 m^2^)	80.8 (26.9)	94.6 (31.0)	68.7 (21.1)	<0.001
Cholesterol (mmol/L)	4.8 (1.2)	5.0(1.3)	4.7 (1.1)	<0.001
Triglycerides (mmol/L)	1.9 (1.9)	2.1 (2.8)	1.7 (1.1)	0.001
LDL-Cholesterol (mmol/L)	2.7 (0.9)	2.9 (1.0)	2.5 (0.9)	<0.001
HDL-Cholesterol (mmol/L)	1.3 (0.4)	1.2 (0.4)	1.3 (0.4)	0.017
BUN(mg/dl)	17.3 (9.0)	15.7 (9.7)	18.9 (8.5)	<0.001
Creatinine(mg/dl)	1.1 (0.8)	1.0 (1.0)	1.1 (0.5)	0.309
SUA(mg/dl)	5.7 (1.6)	5.4 (1.7)	6.0 (1.6)	<0.001

NHANES, National Health and Nutrition Examination Survey; CVD, cardiovascular disease; CKD, chronic kidney disease; HOMA-IR, homeostatic model assessment of insulin resistance; eGFR, estimated glomerular filtration rate.

The values are weighted means (SDs) for continuous variables or numbers (weighted %) for categorical variables.

### Associations of glycemic control with all-cause mortality, CVD-related mortality, diabetes-related mortality and cancer-related mortality

In the fully adjusted model, the adjusted HRs for early-onset participants with T2D in the poorly controlled group were 2.00 (95% CI, 1.30–3.09; P = 0.002) for all-cause mortality, 10.04 (95% CI, 2.57–39.32; P = 0.001) for diabetes-related mortality, 1.81 (95% CI, 0.85–3.85; P = 0.125) for CVD-related mortality and 0.52 (95% CI, 0.12–2.23; P = 0.377) for cancer-related mortality, compared with those in the optimal controlled group.

Compared with those in the optimal controlled group, the adjusted HRs for late-onset participants with T2D in the poorly controlled group were 0.87 (95% CI, 0.54–140; P = 0.561) for all-cause mortality, 1.24 (95% CI, 0.33–4.67; P = 0.751) for diabetes-related mortality, 0.64 (95% CI, 0.30–1.38; P = 0.252) for CVD-related mortality and 1.16 (95% CI, 0.33–4.06; P = 0.815) for cancer-related mortality (Table 2). In addition, CVD and diabetes were the two main causes of death in early-onset T2D patients, and CVD and cancer were the two main causes of death in late-onset T2D patients (S2 Fig).

**Table 2:** HRs (95% CIs) for all-cause and specific mortality according to HbA1c levels among participants in NHANES 1999–2018 (n = 2,831).

	
	Optimal controlled (<7.0%)	Moderately controlled (7.0-8.9%)		Poorly controlled (≥9.0%)	
Early-onset (n = 1,119)					
Nonadjusted					
All-cause	1.00 (refrence)	1.28 (0.77, 2.11)	0.340	1.41 (0.96, 2.06)	0.077
CVD-cause	1.00 (refrence)	1.09 (0.47, 2.53)	0.842	1.29 (0.65, 2.56)	0.460
Cancer-cause	1.00 (refrence)	0.84 (0.18, 3.91)	0.821	0.43 (0.10, 1.96)	0.276
Diabetes-cause	1.00 (refrence)	1.33 (0.38, 4.64)	0.651	4.96 (1.41, 17.5)	0.013
Adjusted ^a^					
All-cause	1.00 (refrence)	1.27 (0.77, 2.12)	0.352	2.00 (1.30, 3.09)	0.002
CVD-cause	1.00 (refrence)	1.30 (0.57, 2.94)	0.535	1.81 (0.85, 3.85)	0.125
Cancer-cause	1.00 (refrence)	0.80 (0.26, 2.41)	0.685	0.52 (0.12, 2.23)	0.377
Diabetes-cause	1.00 (refrence)	1.06 (0.20, 5.68)	0.942	10.04 (2.57, 39.32)	0.001
	Late-onset (n = 1,712)				
Nonadjusted					
All-cause	1.00 (refrence)	1.17 (0.95, 1.45)	0.148	0.84 (0.55, 1.29)	0.428
CVD-cause	1.00 (refrence)	1.20 (0.87, 1.67)	0.268	0.67 (0.35, 1.29)	0.234
Cancer-cause	1.00 (refrence)	0.99 (0.60, 1.63)	0.967	0.73 (0.21, 2.60)	0.629
Diabetes-cause	1.00 (refrence)	1.60 (0.80, 3.23)	0.187	1.65 (0.59, 4.58)	0.337
Adjusted ^a^					
All-cause	1.00 (refrence)	1.20 (0.95, 1.52)	0.128	0.87 (0.54, 1.40)	0.561
CVD-cause	1.00 (refrence)	1.09 (0.75, 1.58)	0.651	0.64 (0.30, 1.38)	0.252
Cancer-cause	1.00 (refrence)	0.97 (0.55, 1.69)	0.901	1.16 (0.33, 4.06)	0.815
Diabetes-cause	1.00 (refrence)	1.58 (0.77, 3.23)	0.212	1.24 (0.33, 4.67)	0.751

NHANES, National Health and Nutrition Examination Survey; HR, hazard ratio; CI, confidence interval; n, number; CVD, cardiovascular disease.

^a^Model: data were adjusted for age, sex, race, education, body mass index, duration of diabetes, hypertension, smoking, drinking, physical activity, diabetes mellitus treatment, complications (retinopathy, CVD, cancer), ALT, AST, BUN, SUA, TG, TC, HDL, LDL, CRP.

**Fig 1:**
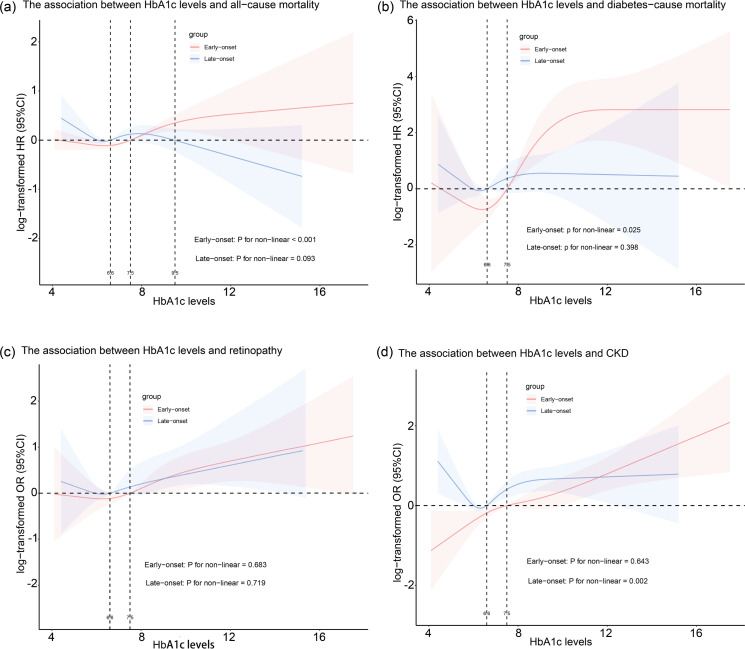
Associations between HbA1c levels and mortality and diabetes complications. A: Restricted cubic spline (RCS) curves showing the relationship between HbA1c levels and the risk of all-cause mortality, with shaded areas indicating 95% confidence intervals. Notably, the risk of death increases sharply when HbA1c is < 6.6% in patients with late-onset T2D. B: RCS curves for diabetes-related mortality. Note that the risk of death increases sharply when HbA1c levels exceed 9%. C: RCS curves for retinopathy risk. The risk increases steadily with increasing HbA1c levels, with no clear inflection point. D: RCS curves for CKD risk. The risk decreases as HbA1c levels drop below 6.6%, but this trend should be interpreted with caution due to wide confidence intervals. HR, hazard ratio; OR, odds ratio; CI, confidence interval. The models were adjusted for age; sex; race; education; body mass index; duration of diabetes; hypertension; smoking status; drinking status; physical activity; diabetes treatment; and ALT, AST, BUN, SUA, TG, TC, HDL, LDL, and CRP levels.

We also used the RCS to flexibly model and visualize the relationships between HbA1c levels and all-cause mortality and diabetes-related mortality in individuals with early- and late-onset T2D (Fig 1). In the early-onset group, the risk of all-cause mortality remained relatively stable when HbA1c levels were less than 6.6%. However, when HbA1c levels exceeded 6.6%, the risk of all-cause mortality began to increase. Interestingly, in the late-onset T2D group, there seemed to be a negative correlation between HbA1c levels and the risk of all-cause mortality when HbA1c levels were below 6.6%; as HbA1c levels increased above 8.0%, the risk of all-cause mortality appeared to decline. Notably, however, this trend did not reach statistical significance (Fig 1A). In the early-onset T2D group, a negative correlation between HbA1c levels and the risk of diabetes-related mortality was observed when HbA1c levels were less than 6.6%. However, this trend did not reach statistical significance. When HbA1c levels ranged between 6.6% and 12%, there was a positive correlation between HbA1c levels and the risk of mortality due to diabetes. However, once HbA1c levels surpassed 12%, the risk of mortality due to diabetes remained relatively stable. In the late-onset T2D group, there appeared to be a negative correlation between HbA1c levels and the risk of mortality related to diabetes when HbA1c levels were less than 6.6%. When HbA1c levels ranged between 6.6% and 8.0%, there seemed to be a positive correlation between HbA1c levels and the risk of mortality related to diabetes. Once HbA1c levels exceeded 8.0%, the risk of diabetes-related mortality appeared to remain relatively stable. However, this trend did not reach statistical significance (Fig 1B).

### Sensitivity and stratified analyses

The sensitivity analyses excluded participants with pancreatic diseases and cancers at the baseline examination (S3 Table), participants who died within 2 years of follow-up (S4 Table), participants who had a history of anemia at baseline (S5 Table) and participants who had a history of CVD (S6 Table). The associations did not substantially change in the different sensitivity analysis models.

Stratified analyses revealed a significant interaction effect between HbA1c levels and CKD status in relation to all-cause mortality among T2D patients (P-interaction = 0.03). Among participants with CKD, compared with those in the poorly controlled group, the adjusted HRs for all-cause mortality were 1.41 (95% CI, 0.65–3.06) in the optimal controlled group and 1.00 (95% CI, 0.50–1.98) in the moderately controlled group. Among participants without CKD, compared with those in the poorly controlled group, the HRs were 0.37 (95% CI, 0.19–0.72) in the optimal controlled group and 0.40 (95% CI, 0.19–0.83) in the moderately controlled group (Table 3). This interaction pattern suggests differential effects of glycemic control on mortality risk on the basis of CKD status. Specifically, optimal glycemic control appears to confer substantial mortality benefits in non-CKD patients, while its protective effect may be attenuated in CKD patients.

**Table 3:** Stratified analyses of the associations (HRs, 95% CIs) between HbA1c levels and all-cause mortality among participants from NHANES 1999--2018 (n = 2,831).

	Optimal controlled (<7.0%)	Moderately controlled (7.0-8.9%)	Poorly controlled (≥9.0%)	*P* for interaction
Age, y				
<60	0.60 (0.35, 1.05)	0.65 (0.37, 1.15)	1.00 (reference)	0.832
≥60	0.23 (0.13, 0.43)	0.48 (0.24, 0.93)	1.00 (reference)	
Gender				
Male	0.68 (0.37, 1.26)	0.60 (0.30, 1.22)	1.00 (reference)	0.323
Female	0.44 (0.24, 0.78)	0.73 (0.43, 1.25)	1.00 (reference)	
Race				
Non-Hispanic White	0.36 (0.18, 0.73)	0.61 (0.26, 1.40)	1.00 (reference)	0.652
Other Race	0.68 (0.38, 1.23)	0.60 (0.35, 1.03)	1.00 (reference)	
Duration of diabetes, year				
<5Y	1.19 (0.20, 6.96)	1.89 (0.18, 19.96)	1.00 (reference)	0.365
5-10Y	0.22 (0.04, 1.33)	0.02 (0.01, 0.30)	1.00 (reference)	
≥10Y	0.89 (0.55, 1.44)	0.93 (0.54, 1.60)	1.00 (reference)	
Weight status, kg/m^2^				
<25	0.13 (0.02, 0.82)	0.51 (0.17, 1.52)	1.00 (reference)	0.471
25 to < 30	1.28 (0.38, 4.32)	0.99 (0.16, 6.23)	1.00 (reference)	
≥30	0.60 (0.30, 1.17)	0.77 (0.47, 1.27)	1.00 (reference)	
Hypertension				
Yes	0.58 (0.37, 0.92)	0.72 (0.43, 1.20)	1.00 (reference)	0.268
No	0.21 (0.05, 0.95)	0.45 (0.13, 1.51)	1.00 (reference)	
Smoking status				
Never	0.42 (0.19, 0.92)	0.79 (0.34, 1.83)	1.00 (reference)	0.510
Ever	0.46 (0.19, 1.13)	0.39 (0.16, 0.93)	1.00 (reference)	
Current	0.99 (0.43, 2.28)	1.10 (0.44, 2.76)	1.00 (reference)	
Alcohol (drinks/day)				
0	0.46 (0.29, 0.71)	0.86 (0.52, 1.42)	1.00 (reference)	0.103
1-2	1.81 (0.53, 6.20)	1.22 (0.28, 5.32)	1.00 (reference)	
>2	0.26 (0.08, 0.85)	0.31 (0.11, 0.90)	1.00 (reference)	
Physical activity				
Low intensity	0.70 (0.37, 1.34)	0.61 (0.34, 1.12)	1.00 (reference)	0.634
Moderate intensity	0.21 (0.06, 0.70)	0.25 (0.07, 0.84)	1.00 (reference)	
High intensity	0.09 (0.02, 0.52)	0.58 (0.14, 2.38)	1.00 (reference)	
Chronic kidney disease				
Yes	1.41 (0.65, 3.06)	1.00 (0.50, 1.98)	1.00 (reference)	0.030
No	0.37 (0.19, 0.72)	0.40 (0.19, 0.83)	1.00 (reference)	
Diabetes mellitus treatment				
Insulin	0.34 (0.13, 0.89)	0.34 (0.13, 0.86)	1.00 (reference)	0.925
Insulin+oral medications	0.28 (0.08, 0.95)	1.20 (0.51, 2.83)	1.00 (reference)	
Oral medications	0.66 (0.39, 1.11)	0.52 (0.25, 1.09)	1.00 (reference)	
No pharmacotherapy	1.67 (0.35, 8.04)	3.20 (0.41, 25.33)	1.00 (reference)	

NHANES, National Health and Nutrition Examination Survey; HR, hazard ratio; CI, confidence interval; n, number; Data were adjusted for age, sex, race, education, body mass index, duration of diabetes, hypertension, smoking, drinking, physical activity, diabetes mellitus treatment, complications (retinopathy, CVD, cancer), ALT, AST, BUN, SUA, TG, TC, HDL, LDL, CRP.

### The associations of glycemic control with the risk of retinopathy, CKD and cardiovascular disease

According to the fully adjusted model, the adjusted ORs for early-onset participants with T2D in the poorly control group were 1.80 (95% CI, 1.10–2.94; P = 0.022) for retinopathy, 2.54 (95% CI, 1.65–3.92; P < 0.001) for CKD, and 1.00 (95% CI, 0.57–1.77; P = 0.996) for CVD, compared with the optimal control group; the adjusted ORs for late-onset participants with T2D in the poorly control group were 2.12 (95% CI, 1.32–3.41; P = 0.002) for retinopathy, 2.30 (95% CI, 1.45–3.63; P = 0.001) for CKD, and 0.53 (95% CI, 0.28–1.01; P = 0.055) for CVD, compared with the optimal control group (Table 4).

**Table 4:** ORs (95% CIs) for diabetic complications according to HbA1c levels among participants in NHANES 1999--2018 (n = 2,831).

	
	Optimal controlled (<7.0%)	Moderately controlled (7.0-8.9%)		Poorly controlled (≥9.0%)	
Early-onset (n = 1,119)					
Nonadjusted					
Retinopathy	1.00 (refrence)	1.19 (0.79, 1.80)	0.396	1.75 (1.16, 2.64)	0.009
CKD	1.00 (refrence)	1.39 (0.98, 1.97)	0.070	2.41 (1.67, 3.49)	<0.001
CVD	1.00 (refrence)	1.40 (0.85, 2.30)	0.191	0.96 (0.58, 1.59)	0.880
Adjusted ^a^					
Retinopathy	1.00 (refrence)	1.01 (0.64, 1.59)	0.976	1.8 (1.1, 2.94)	0.022
CKD	1.00 (refrence)	1.25 (0.86, 1.80)	0.240	2.54 (1.65, 3.92)	<0.001
CVD	1.00 (refrence)	1.07 (0.65, 1.78)	0.782	1.00 (0.57, 1.77)	0.996
	Late-onset (n = 1,712)				
Nonadjusted					
Retinopathy	1.00 (refrence)	1.36 (1.01, 1.83)	0.043	2.18 (1.41, 3.38)	<0.001
CKD	1.00 (refrence)	1.49 (1.09, 2.04)	0.013	2.12 (1.28, 3.50)	0.004
CVD	1.00 (refrence)	0.83 (0.62, 1.11)	0.214	0.57 (0.33, 0.99)	0.047
Adjusted ^a^					
Retinopathy	1.00 (refrence)	1.23 (0.89, 1.69)	0.212	2.12 (1.32, 3.41)	0.002
CKD	1.00 (refrence)	1.35 (0.98, 1.85)	0.070	2.30 (1.45, 3.63)	0.001
CVD	1.00 (refrence)	0.77 (0.56,1.07)	0.125	0.53 (0.28,1.01)	0.055

NHANES, National Health and Nutrition Examination Survey; OR, odds ratio; CI, confidence interval; n, number; CKD, chronic kidney disease; CVD, cardiovascular disease.

^a^Model: Data were adjusted for age; sex; race; education; body mass index; duration of diabetes; hypertension; smoking; drinking; physical activity; diabetes mellitus treatment; and ALT, AST, BUN, SUA, TG, TC, HDL, LDL, and CRP levels.

We also used RCS to flexibly model and visualize the relationships between HbAlc and the risk of retinopathy and CKD in the early- and late-onset T2D groups. There was a positive correlation between HbAlc levels and the risk of retinopathy in the late-onset T2D group (Fig 1C). Furthermore, in the early-onset T2D group, there was a positive correlation between HbA1c levels and the risk of CKD. In contrast, in the late-onset T2D group, a negative correlation between HbA1c levels and the risk of CKD occurred when HbA1c levels were less than 6.6%. For HbA1c levels between 6.6% and 9.0%, there was a positive correlation between HbA1c levels and the risk of CKD. However, when HbA1c levels were above 9.0%, the risk of CKD remained relatively stable (Fig 1D).

## Discussion

In this large-scale retrospective study, we found that in individuals with early-onset T2D, optimal glycemic control was associated with a reduced risk of all-cause mortality and diabetes-related mortality, without any significant association with mortality from CVD or cancer. However, in individuals with late-onset T2D, there was no evidence of an association between glycemic control and all-cause mortality or death from other causes. Logistic analysis revealed that optimal glycemic control was also linked to lower risks of retinopathy and CKD in both individuals with early-onset and late-onset T2D, but it did not have a significant effect on CVD risk. To summarize, optimal glycemia control was associated with a reduced risk of all-cause mortality, diabetes-related mortality, retinopathy, and CKD in individuals with early-onset T2D, whereas in individuals with late-onset T2D, optimal glycemia control was found to be linked only to lower risks of retinopathy and CKD.

Although numerous studies have investigated the relationship between strict glycemic control and the risk of mortality or complications of diabetes, a consistent conclusion has yet to be reached. On the basis of a prospective cohort study derived from the United Kingdom Prospective Diabetes Study (UKPDS), participants with T2D who underwent strict glycemic control had a lower risk of microvascular complications and all-cause mortality than those receiving conventional dietary therapy did, as observed during a 10-year posttrial follow-up period [29]. Surprisingly, during the 15-year posttrial follow-up period, conflicting results emerged, with no evidence of a legacy effect or benefit in terms of reduced mortality with intensive glucose control [30]. In the study by [30], the mean age at diagnosis of diabetes participants was approximately 50 years, and the mean duration of disease was approximately 10 years. The participants were not early-onset T2D.

According to our study, optimal glycemic control was not associated with the risk of all-cause mortality, including mortality related to CVD, diabetes and cancer, in individuals with late-onset T2D. Additionally, RCS analysis yielded consistent results. Notably, the RCS analysis results further demonstrated that an HbA1c<6.6% was associated with an increased risk of all-cause mortality. In our study, the individuals with late-onset T2D had a mean age of 73.5 (±6.2) years and a mean disease duration of 6.6 (±5.4) years. Among the participants, 29.8% reported macrovascular complications, such as heart failure, coronary heart disease, angina, and myocardial infarction, whereas 23.3% reported malignant cancers. Additionally, 34.8% had CKD (eGFR < 60 ml/min/1.73 m^2^). These findings further suggest that participants with diabetes who are more than 60 years old are often complicated with complex medical problems, which may limit the benefit they derive from strict glucose control strategies. Furthermore, aggressive measures to achieve lower HbA1c levels may result in poor clinical outcomes [31].

Our study revealed that glycemic control was not associated with all-cause mortality risk in individuals with late-onset T2D, which is consistent with existing evidence that aggressive glycemic targets may not be appropriate for older adults with multiple comorbidities [12]. The American Diabetes Association (ADA) Standards of Care explicitly recommend less stringent targets (e.g., HbA1c < 8.0%) in this population to avoid hypoglycemia and polypharmacy risks, particularly when life expectancy is limited [19]. The absence of an association with late-onset T2D may reflect competing risks of nondiabetic mortality (e.g., cancer, frailty-related outcomes) that dominate in older adults, diluting the long-term benefits of intensive glycemic control [32,33]. For clinicians managing late-onset T2D, these results reinforce the need to prioritize individualized goals over uniform HbA1c thresholds.

In contrast to our findings in individuals with late-onset T2D, our research indicated that optimal glycemic control was significantly associated with reduced risks of all-cause mortality and diabetes-related mortality in individuals with early-onset T2D. Specifically, the optimal glycemic control group presented a significant 100% reduction in the risk of all-cause mortality and a notable 904% decrease in the risk of diabetes-related mortality. However, optimal glycemic control was not associated with a reduced risk of CVD or cancer mortality in these patients. Among individuals with early-onset T2D, the mean age was 48.2 (±13.5) years, and the mean duration of the disease was 17.1 (±13.9) years. The prevalence rates of cardiovascular complications (18.8%) and cancer complications (7.1%) were lower than those in individuals with late-onset disease. The early-onset cohort had a longer duration of disease, experienced fewer complications, and presented a longer life expectancy than did the late-onset cohort did, which was likely attributable to their younger age at diagnosis and younger mean age at study completion. The lack of association between early-onset T2D and CVD or cancer-related morbidity/mortality in our analysis may reflect the younger age of this cohort at the end of follow-up. To fully evaluate their long-term risk for these age-driven outcomes, extended follow-up until participants reach comparable ages to those in the late-onset group is needed.

Importantly, diabetes ranks as the second leading cause of death in individuals with early-onset T2D, highlighting the importance of effective glycemic management strategies in this population. These findings suggest that optimal glycemic control, as defined by an HbA1c level below 7%, may be appropriate for younger, relatively healthy patients with longer life expectancies. However, there is currently very little research on the benefits of optimal glucose control in individuals with early-onset T2D. On the basis of a few studies, we can conclude that early-onset T2D patients may receive greater net benefits from optimal glucose control interventions than late-onset T2D patients do [34,35]. For example, for a patient in whom diabetes developed before 50 years of age, reducing HbA1c levels from 9% to 7% results in an estimated 2.3% decrease in the risk of blindness due to retinopathy. Similarly, a patient with diabetes onset at 65 years of age is expected to have a 0.5% reduction in the risk of blindness [36]. Therefore, in individuals with early-onset T2D, the benefits of glucose control are more likely to materialize.

Consistent with our experimental results, CVD mortality and complications accounted for the most frequently occurring adverse events and remained the primary cause of mortality in the diabetes population. In addition, there was no correlation between glycemic control and cardiovascular mortality or complications in individuals with early-onset or late-onset T2D. These results also support the hypothesis that hyperglycemia may not be the primary cause of CVD in these individuals [37]. This further emphasizes the complex and unclear etiology of CVD associated with diabetes. Although the management of individuals with T2D has undergone a major conceptual change, with treatment objectives shifting to include cardiocentric goals in the subpopulation with high cardiovascular risk. Emerging evidence highlights that newer antidiabetic agents, such as SGLT2 inhibitors (SGLT2is), GLP-1 receptor agonists (GLP-1 s), and GIP/GLP-1 receptor agonists, demonstrate significant cardiovascular benefits, including reduced risks of heart failure, atherosclerotic events, and mortality in patients with T2D [38,39]. Analysis of the 2017–2018 NHANES cycle revealed limited utilization of newer antihyperglycemic agents, with only 7.1% of participants receiving either SGLT2 inhibitors or GLP-1 receptor agonists, despite their established cardiorenal benefits and guideline recommendations for high-risk populations [40]. As such, our findings may not fully reflect the potential CVD risk reduction achievable with contemporary treatment strategies. Future studies should account for the evolving use of these therapies, as their increasing prevalence may substantially alter longitudinal outcomes in T2D, particularly in early-onset populations.

There are several limitations to the current study. First, the reliance on self-reported diabetes diagnoses and retrospective HbA1c measurements in the NHANES may introduce misclassification bias. We excluded participants with missing glycemic data and restricted the early-onset T2D population to individuals aged 18–39 years to minimize the incidence of T1D. Second, the cross-sectional design limits our ability to establish temporal relationships between glycemic control and long-term outcomes. Third, the NHANES was not specifically designed to evaluate the effects of glucose control, and variables not captured by the survey (e.g., medication adherence, lifestyle changes) could have influenced outcomes.

In conclusion, in a nationally representative sample of U.S. adults, we showed that in individuals with early-onset T2D, optimal glycemic control was associated with a reduced risk of all-cause mortality and diabetes-related mortality, with no significant association with mortality caused by CVD or cancer. Moreover, logistic analysis demonstrated that optimal glycemic control was associated with lower risks of retinopathy and CKD in both individuals with early- and late-onset T2D, but it was not significantly associated with CVD risk. In contrast, in individuals with late-onset T2D, there was no evidence of an association between glycemic control and all-cause mortality or death from other causes. Thus, optimal glycemic control appears to be associated with a reduced risk of all-cause mortality, diabetes-related mortality, retinopathy, and CKD in individuals with early-onset T2D; in individuals with late-onset T2D, the associations were limited to lower risks of retinopathy and CKD.

### Consent for publication

All the authors had access to the data in the study and accept responsibility for submitting the manuscript for publication.

## Supporting information

S1 Fig Differences in glucose control between individuals with early-onset and late-onset T2D in NHANES (1999–2018). (DOCX)

S2 Fig Differences in the proportion of deaths between early-onset and late-onset T2D patients in NHANES (1999–2018). (DOCX)

S1 Table Basic demographic, behavioral, biochemical profile and complications characteristics of early-onset T2D in NHANES (1999–2018). (DOCX)

S2 Table Basic demographic, behavioral, biochemical profile and complications characteristics of late-onset T2D in NHANES (1999–2018). (DOCX)

S3 Table HR (95% CIs) for all-cause and cause-specific mortality according to HbA1c levels among participants after excluding patients with cancer (n = 2,352). (DOCX)

S4 Table HR (95% CIs) for all-cause and cause-specific mortality according to HbA1c levels among participants after excluding excluding subjects who died within two years of follow-up (n = 2,665). (DOCX)

S5 Table HR (95% CIs) for all-cause and cause-specific mortality according to HbA1c levels among participants after excluding patients with anemia (n = 2,273). (DOCX)

S6 Table HR (95% CIs) for all-cause and cause-specific mortality according to HbA1c levels among participants after excluding patients with cardiovascular disease (n = 2,111). (DOCX)

## Abbreviations

T2D: Type 2 Diabetes
T1D: Type 1 Diabetes
NHANES: National Health and Nutrition Examination Survey
CVD: Cardiovascular disease
CKD: Chronic kidney disease
HR: Hazard ratio
OR: Odds ratio
CRP: C-reactive protein
RCS: Restricted cubic splines
BUN: Blood urea nitrogen
eGFR: Estimated Glomerular Filtration Rate
BMI: Body mass index
SBP: Systolic blood pressure.
