# Supplementary material for: Divergent impacts of glycemic control on mortality and complications in patients with early-versus late-onset type 2 diabetes: A retrospective cohort study
Source: PLoS One. 2025 May 23;20(5):e0322886. doi: 10.1371/journal.pone.0322886 (PMC12101672; doi:10.1371/journal.pone.0322886)

S2 Fig: Differences in the proportion of deaths between early-onset and late-onset T2D patients in NHANES (1999-2018)


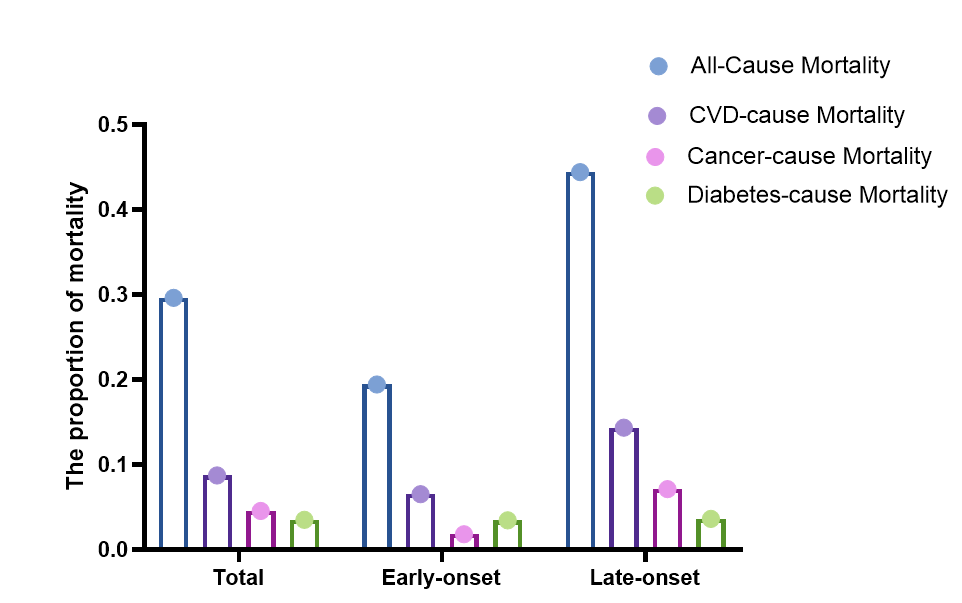

Supplement: S2 Fig — (DOCX) [file pone.0322886.s002.docx]
