# Supplementary material for: Divergent impacts of glycemic control on mortality and complications in patients with early-versus late-onset type 2 diabetes: A retrospective cohort study
Source: PLoS One. 2025 May 23;20(5):e0322886. doi: 10.1371/journal.pone.0322886 (PMC12101672; doi:10.1371/journal.pone.0322886)
Supplement: S1 Table — (DOCX) [file pone.0322886.s003.docx]

| **S1 Table:** Basic demographic, behavioral, biochemical profile and complications characteristics of early-onset T2D in NHANES (1999-2018) | | | | | |
| --- | --- | --- | --- | --- | --- |
|  | Early-onset  T2D | Optimal control  (<7.0%) | Moderately control  (7.0-8.9%) | Poorly control  (≥9.0%) | *P* |
| Participants, n | 1119 | 432 | 374 | 313 |  |
| Characteristic |  |  |  |  |  |
| Gender, n(%) |  |  |  |  |  |
| Male | 499 (44.6) | 182 (42.1) | 169 (45.2) | 148 (47.3) | 0.362 |
| Female | 620 (55.4) | 250 (57.9) | 205 (54.8) | 165 (52.7) |  |
| Age, years, mean(SD) | 48.2 (13.5) | 47.4 (14.5) | 50.8 (13.4) | 46.2(11.7) | <0.001 |
| Age at diagnosis, mean (SD) | 31.1 (6.2) | 31.2(6.5) | 31.0 (6.0) | 31.1 (5.9) | 0.904 |
| BMI , mean (SD) | 33.8 (8.5) | 33.6 (8.7) | 34.8 (8.9) | 33.0 (7.6) | 0.02 |
| Weight status , n(%) |  |  |  |  |  |
| Normal: BMI of <25, | 131 (12.1) | 61 (14.5) | 39 (10.9) | 31 (10.2) | 0.184 |
| Overweight: BMI of 25 to <30 | 259 (23.9) | 97 (23.0) | 79 (22.1) | 83 (27.4) |  |
| Obese: BMI of ≥30 | 693 (64.0) | 264 (62.6) | 240 (67.0) | 189 (62.4) |  |
| Race, n(%) |  |  |  |  | 0.013 |
| Mexican American | 257 (23.0) | 85 (19.7) | 90 (24.1) | 82 (26.2) |  |
| Other Hispanic | 121 (10.8) | 43 (10.0) | 43 (11.5) | 35 (11.2) |  |
| Non-Hispanic White | 311 (27.8) | 140 (32.4) | 101 (27.0) | 70 (22.4) |  |
| Non-Hispanic Black | 318 (28.4) | 110 (25.5) | 105 (28.1) | 103 (32.9) |  |
| Other Race | 112 (10.0) | 54 (12.5) | 35 ( 9.4) | 23 (7.3) |  |
| Eduactional level, n (%) |  |  |  |  | 0.012 |
| High school or less | 633 (56.6) | 225 (52.1) | 206 (55.1) | 202 (64.5) |  |
| Some college | 338 (30.2) | 144 (33.3) | 113 (30.2) | 81 (25.9) |  |
| College graduate | 148 (13.2) | 63 (14.6) | 55 (14.7) | 30 ( 9.6) |  |
| Insurance, n(%) |  |  |  |  | 0.002 |
| Any insurance | 882 (78.7) | 338 (78.2) | 315 (84.1) | 229 (73.2) |  |
| Uninsured | 237 (21.3) | 94 (21.8) | 59 (15.9) | 84 (26.8) |  |
| Hypentension, n(%) | 718 (64.2) | 269 (62.3) | 255 (68.2) | 194 (62.0) | 0.139 |
| Smoking, n(%) | 273 (24.4) | 113 (26.2) | 100 (26.7) | 60 (19.2) | 0.144 |
| Alcohol(drinks/day), n(%) |  |  |  |  |  |
| 0 | 536 (47.9) | 194 (44.9) | 187 (50.0) | 155 (49.5) | 0.239 |
| 1- 2 | 354 (31.6) | 151 (35.0) | 116 (31.0) | 87 (27.8) |  |
| >2 | 229 (20.5) | 87 (20.1) | 71 (19.0) | 71 (22.7) |  |
| Physical activity, n(%) |  |  |  |  |  |
| Low intensity | 525 (46.9) | 200 (46.3) | 178 (47.6) | 147 (47.0) | 0.972 |
| Moderate-intensity | 337 (30.1) | 128 (29.6) | 113 (30.2) | 96 (30.7) |  |
| High-intensity | 257 (23.0) | 104 (24.1) | 83 (22.2) | 70 (22.4) |  |
| The duration of diabetes,  mean (SD) | 17.1 (13.9) | 16.2 (15.3) | 19.8 (13.9) | 15.1 (11.2) | <0.001 |
| Comorbidities, n(%) |  |  |  |  |  |
| Retinopathy | 335 (30.0) | 106 (24.7) | 120 (32.1) | 109 (34.9) | 0.017 |
| CVD | 209 (18.7) | 78 (18.1) | 81 (21.7) | 50 (16.0) | 0.195 |
| Tumor | 80 (7.1) | 38 (8.8) | 22 (5.9) | 20 (6.4) | 0.23 |
| Biochemical profile, mean(SD) |  |  |  |  |  |
| Glucose (mmol/L) | 10.1 (4.8) | 6.6 (1.8) | 9.5 (3.2) | 14.8 (5.0) | <0.001 |
| HbA1c(% ) | 8.0 (2.2) | 6.0 (0.6) | 7.9 (0.6) | 11.0 (1.6) | <0.001 |
| HOMA-IR | 11.1 (18.6) | 6.6 (9.0) | 11.5 (15.7) | 15.9 (26.7) | <0.001 |
| C-reactive protein(mg/dL) | 3.0 (6.8) | 2.8 (7.1) | 3.2 (6.8) | 2.9 (6.3) | 0.722 |
| ALT(U/I) | 28.4 (46.1) | 27.3 (23.4) | 26.4 (18.8) | 32.3 (80.2) | 0.215 |
| AST(U/I) | 26.0 (25.6) | 26.4 (21.8) | 25.1 (16.4) | 26.6 (37.1) | 0.688 |
| eGFR(ml/min/1.73 m^2^) | 94.6 (31.0) | 92.6 (31.7) | 90.4 (31.5) | 102.3 (27.9) | <0.001 |
| Cholesterol (mmol/L) | 5.0 (1.3) | 4.8 (1.3) | 4.8 (1.2) | 5.5 (1.5) | <0.001 |
| Triglycerides (mmol/L) | 2.1(2.8) | 1.5 (0.7) | 2.2 (3.8) | 2.7 (2.9) | <0.001 |
| LDL-Cholesterol (mmol/L) | 2.9 (1.0) | 2.8 (1.1) | 2.6 (0.9) | 3.2 (1.1) | <0.001 |
| HDL-Cholesterol (mmol/L) | 1.2 (0.4) | 1.3 (0.4) | 1.2 (0.4) | 1.2 (0.3) | 0.013 |
| BUN(mg/dl) | 15.7 (9.7) | 15.4 (9.4) | 16.8 (11.5) | 14.8 (7.3) | 0.021 |
| Creatinine(mg/dl) | 1.0 (1.0) | 1.1 (1.2) | 1.1 (1.0) | 0.9 (0.6) | 0.003 |
| SUA(mg/dl) | 5.4 (1.7) | 5.7 (1.6) | 5.5 (1.8) | 5.0 (1.5) | <0.001 |
| NHANES, National Health and Nutrition Examination Survey; CVD, cardiovascular disease; CKD, chronic kidney disease; HOMA-IR, homeostatic model assessment of insulin resistance; eGFR, estimated glomerular filtration rate;  Values are weighted mean (SD) for continuous variables or numbers (weighted %) for categorical variables. | | | | | |
