# Supplementary material for: Divergent impacts of glycemic control on mortality and complications in patients with early-versus late-onset type 2 diabetes: A retrospective cohort study
Source: PLoS One. 2025 May 23;20(5):e0322886. doi: 10.1371/journal.pone.0322886 (PMC12101672; doi:10.1371/journal.pone.0322886)
Supplement: S2 Table — (DOCX) [file pone.0322886.s004.docx]

| **S2 Table:** Basic demographic, behavioral, biochemical profile and complications characteristics of late-onset T2D in NHANES (1999-2018). | | | | | |
| --- | --- | --- | --- | --- | --- |
|  | Late-onset  T2D | Optimal control  (<7.0%) | Moderately control  (7.0-8.9%) | Poorly control  (≥9.0%) | *P* |
| Participants, n | 1712 | 1070 | 508 | 134 |  |
| Characteristic |  |  |  |  |  |
| Gender, n(%) |  |  |  |  |  |
| Male | 933 (54.5) | 579 (54.1) | 282 (55.5) | 72 (53.7) | 0.858 |
| Female | 779 (45.5) | 491 (45.9) | 226 (44.5) | 62 (46.3) |  |
| Age, years, mean(SD) | 73.5 (6.2) | 73.5 (6.2) | 73.8 (6.2) | 72.0 (6.2) | 0.011 |
| Age at diagnosis, mean (SD) | 73.5 (6.2) | 67.4 (6.2) | 66.0(5.8) | 65.8 (5.7) | 0.525 |
| BMI , mean (SD) | 30.3 (5.9) | 30.2(6.1) | 30.2 (5.6) | 30.3 (5.9) | 0.988 |
| Weight status , n(%) |  |  |  |  |  |
| Normal: BMI of <25, | 280 (17.0) | 176 (17.2) | 81 (16.5) | 23 (17.6) | 0.544 |
| Overweight: BMI of 25 to <30 | 600 (36.4) | 388 (37.8) | 166 (33.9) | 46 (35.1) |  |
| Obese: BMI of ≥30 | 767 (46.6) | 462 (45.0) | 243 (49.6) | 62 (47.3) |  |
| Race, n(%) |  |  |  |  |  |
| Mexican American | 283 (16.5) | 171 (16.0) | 84 (16.5) | 28 (20.9) | 0.089 |
| Other Hispanic | 118 (6.9) | 73 (6.8) | 33 (6.5) | 12 (9.0) |  |
| Non-Hispanic White | 819 (47.8) | 528 (49.3) | 244 (48.0) | 47 (35.1) |  |
| Non-Hispanic Black | 345 (20.2) | 212 (19.8) | 96 (18.9) | 37 (27.6) |  |
| Other Race | 147 (8.6) | 86 (8.0) | 51 (10.0) | 10 (7.5) |  |
| Eduactional level, n (%) |  |  |  |  |  |
| High school or less | 1103 (64.4) | 675 (63.1) | 334 (65.7) | 94 (70.1) | 0.116 |
| Some college | 373 (21.8) | 235 (22.0) | 107 (21.1) | 31 (23.1) |  |
| College graduate | 236 (13.8) | 160 (15.0) | 67 (13.2) | 9 (6.7) |  |
| Insurance, n(%) |  |  |  |  |  |
| Any insurance | 1642 (96.0) | 1029 (96.3) | 486 (95.7) | 127 (94.8) | 0.518 |
| Uninsured | 67 (3.9) | 39 (3.7) | 21 (4.1) | 7 (5.2) |  |
| Hypentension, n(%) | 1374 (80.3) | 879 (82.1) | 393 (77.4) | 102 (76.1) | 0.038 |
| Smoking, n(%) | 736 (43.0) | 478 (44.7) | 203 (40.0) | 55 (41.0) | 0.153 |
| Alcohol(drinks/day), n(%) |  |  |  |  |  |
| 0 | 1078 (63.0) | 657 (61.4) | 337 (66.3) | 84 (62.7) | 0.258 |
| 1- 2 | 535 (31.2) | 351 (32.8) | 145 (28.5) | 39 (29.1) |  |
| >2 | 99 (5.8) | 62 (5.8) | 26 (5.1) | 11 (8.2) |  |
| Physical activity, n(%) |  |  |  |  |  |
| Low intensity | 1004 (58.6) | 625 (58.4) | 292 (57.5) | 87 (64.9) | 0.377 |
| Moderate-intensity | 536 (31.3) | 331 (30.9) | 166 (32.7) | 39 (29.1) |  |
| High-intensity | 172 (10.0) | 114 (10.7) | 50 (9.8) | 8 (6.0) |  |
| The duration of diabetes,  mean (SD) | 6.6 (5.4) | 6.1 (5.3) | 7.8 (5.6) | 6.2 (4.7) | <0.001 |
| Comorbidities, n(%) |  |  |  |  |  |
| Retinopathy | 250 (14.6) | 135 (12.6) | 83 (16.3) | 32 (23.9) | 0.005 |
| CVD | 511 (29.8) | 333 (31.1) | 143 (28.1) | 35 (26.1) | 0.298 |
| Tumor | 399 (23.3) | 264 (24.7) | 113 (22.2) | 22 (16.4) | 0.23 |
| Biochemical profile, mean(SD) |  |  |  |  |  |
| Glucose (mmol/L) | 8.0 (2.8) | 7.0 (1.5) | 9.1 (2.4) | 13.7 (5.0) | <0.001 |
| HbA1c(% ) | 7.0 (1.4) | 6.2 (0.5) | 7.6 (0.5) | 10.5 (1.4) | <0.001 |
| HOMA-IR | 6.9 (10.4) | 5.2 (6.1) | 9.4 (13.2) | 11.3 (20.2) | <0.001 |
| C-reactive protein(mg/dL) | 1.9 (5.6) | 1.8 (5.7) | 2.0 (4.8) | 2.7 (7.6) | 0.342 |
| ALT(U/I) | 22.6 (12.4) | 21.5 (11.1) | 24.1 (13.3) | 26.3 (17.1) | <0.001 |
| AST(U/I) | 24.7 (11.6) | 24.3 (10.1) | 24.9 (11.3) | 27.4 (20.2) | 0.013 |
| eGFR(ml/min/1.73 m^2^) | 68.7 (21.1) | 68.1 (20.8) | 69.2 (21.4) | 71.3 (22.3) | 0.221 |
| Cholesterol (mmol/L) | 4.7 (1.1) | 4.6 (1.1) | 4.7 (1.1) | 5.0 (1.2) | 0.001 |
| Triglycerides (mmol/L) | 1.7 (1.1) | 1.6 (1.0) | 1.9 (1.2) | 1.9 (1.1) | 0.001 |
| LDL-Cholesterol (mmol/L) | 2.51 (0.9) | 2.5 (0.9) | 2.4 (0.8) | 2.9 (1.2) | 0.002 |
| HDL-Cholesterol (mmol/L) | 1.3 (0.4) | 1.3 (0.4) | 1.2 (0.3) | 1.2 (0.4) | <0.001 |
| BUN(mg/dl) | 18.9 (8.5) | 18.9 (8.5) | 19.0 (8.3) | 18.4 (8.8) | 0.801 |
| Creatinine(mg/dl) | 1.1 (0.5) | 1. (0.6) | 1.1 (0.5) | 1.0 (0.4) | 0.44 |
| SUA(mg/dl) | 6.0 (1.6) | 6.0 (1.6) | 5.9 (1.5) | 5.4 (1.8) | <0.001 |
| NHANES, National Health and Nutrition Examination Survey; CVD, cardiovascular disease; CKD, chronic kidney disease; HOMA-IR, homeostatic model assessment of insulin resistance; eGFR, estimated glomerular filtration rate;  Values are weighted mean (SD) for continuous variables or numbers (weighted %) for categorical variables. | | | | | |
