# Supplementary material for: Divergent impacts of glycemic control on mortality and complications in patients with early-versus late-onset type 2 diabetes: A retrospective cohort study
Source: PLoS One. 2025 May 23;20(5):e0322886. doi: 10.1371/journal.pone.0322886 (PMC12101672; doi:10.1371/journal.pone.0322886)
Supplement: S4 Table — (DOCX) [file pone.0322886.s006.docx]

| **S4 Table:** HR (95% CIs) for all-cause and cause-specific mortality according to HbA1c levels among participants after excluding excluding subjects who died within two years of follow-up (n=2,665) | | | | | | | | | | | |
| --- | --- | --- | --- | --- | --- | --- | --- | --- | --- | --- | --- |
|  | Early-onset (n=1,081) | | | | |  | Late-onset (n=1,584) | | | | |
|  | Optimal control  (<7.0%) | Moderately control  (7.0-8.9%) | | Poorly control  (≥9.0%) | |  | Optimal control  (<7.0%) | Moderately control  (7.0-8.9%) | | Poorly control  (≥9.0%) | |
| Non-adjusted |  |  |  |  |  |  |  |  |  |  |  |
| All-cause | 1.00 (refrence) | 1.06 (0.62, 1.81) | 0.827 | 1.53 (1.02, 2.28) | 0.040 |  | 1.00 (refrence) | 1.15 (0.92, 1.44) | 0.214 | 0.75 (0.46, 1.22) | 0.240 |
| CVD-cause | 1.00 (refrence) | 0.82 (0.35, 1.94) | 0.656 | 1.34 (0.64, 2.81) | 0.436 |  | 1.00 (refrence) | 1.18 (0.82, 1.68) | 0.371 | 0.50 (0.22, 1.12) | 0.092 |
| Cancer-cause | 1.00 (refrence) | 0.98 (0.20, 4.83) | 0.980 | 0.51 (0.10, 2.43) | 0.394 |  | 1.00 (refrence) | 1.06 (0.59, 1.90) | 0.852 | 0.70 (0.14, 3.44) | 0.663 |
| Diabetes-cause | 1.00 (refrence) | 1.20 (0.32, 4.56) | 0.788 | 5.31 (1.41, 19.99) | 0.014 |  | 1.00 (refrence) | 1.89 (0.88, 4.10) | 0.105 | 1.72 (0.45, 6.55) | 0.427 |
| Adjusted ^a^ |  |  |  |  |  |  |  |  |  |  |  |
| All-cause | 1.00 (refrence) | 1.03 (0.61, 1.73) | 0.910 | 2.31 (1.44, 3.72) | 0.001 |  | 1.00 (refrence) | 1.16 (0.91, 1.48) | 0.225 | 0.75 (0.44, 1.28) | 0.288 |
| CVD-cause | 1.00 (refrence) | 0.92 (0.39, 2.15) | 0.848 | 2.00 (0.86, 4.67) | 0.109 |  | 1.00 (refrence) | 1.07 (0.72, 1.59) | 0.738 | 0.47 (0.19, 1.18) | 0.106 |
| Cancer-cause | 1.00 (refrence) | 0.90 (0.33, 2.43) | 0.838 | 0.60 (0.06, 5.64) | 0.655 |  | 1.00 (refrence) | 1.11 (0.59, 2.09) | 0.739 | 1.15 (0.25, 5.28) | 0.856 |
| Diabetes-cause | 1.00 (refrence) | 0.93 (0.15, 5.54) | 0.933 | 11.31 (2.62, 48.83) | 0.001 |  | 1.00 (refrence) | 1.66 (0.73, 3.79) | 0.230 | 1.52 (0.32, 7.11) | 0.598 |
| NHANES, National Health and Nutrition Examination Survey; HR, hazard ratio; CI, confidence interval; n, the number; CVD, cardiovascular disease.  ^a^ Model: data were adjusted for adjusted for age, sex, race, education, body mass index, the duration of diabetes, hypertension, smoking, drinking, physical activity, diabetes treatment, complications (retinopathy, CVD, cancer), ALT, AST, BUN, SUA, TG, TC, HDL, LDL, CRP. | | | | | | | | | | | |
