# Supplementary material for: Divergent impacts of glycemic control on mortality and complications in patients with early-versus late-onset type 2 diabetes: A retrospective cohort study
Source: PLoS One. 2025 May 23;20(5):e0322886. doi: 10.1371/journal.pone.0322886 (PMC12101672; doi:10.1371/journal.pone.0322886)
Supplement: S5 Table — (DOCX) [file pone.0322886.s007.docx]

| **S5 Table:** HR (95% CIs) for all-cause and cause-specific mortality according to HbA1c levels among participants after excluding patients with anemia (n=2,273) | | | | | | | | | | | |
| --- | --- | --- | --- | --- | --- | --- | --- | --- | --- | --- | --- |
|  | Early-onset (n=917) | | | | |  | Late-onset (n=1,356) | | | | |
|  | Optimal control  (<7.0%) | Moderately control  (7.0-8.9%) | | Poorly control  (≥9.0%) | |  | Optimal control  (<7.0%) | Moderately control  (7.0-8.9%) | | Poorly control  (≥9.0%) | |
| Non-adjusted |  |  |  |  |  |  |  |  |  |  |  |
| All-cause | 1.00 (refrence) | 1.58 (0.88, 2.83) | 0.126 | 1.77 (1.13, 2.77) | 0.013 |  | 1.00 (refrence) | 1.22 (0.96, 1.56) | 0.107 | 0.78 (0.45, 1.35) | 0.367 |
| CVD-cause | 1.00 (refrence) | 1.32 (0.51, 3.42) | 0.573 | 1.52 (0.70, 3.31) | 0.291 |  | 1.00 (refrence) | 1.28 (0.87, 1.87) | 0.206 | 0.62 (0.27, 1.42) | 0.259 |
| Cancer-cause | 1.00 (refrence) | 1.04 (0.20, 5.40) | 0.962 | 0.53 (0.11, 2.60) | 0.431 |  | 1.00 (refrence) | 1.04 (0.59, 1.85) | 0.890 | 0.91 (0.23, 3.55) | 0.895 |
| Diabetes-cause | 1.00 (refrence) | 1.43 (0.30, 6.73) | 0.651 | 6.59 (1.44, 30.14) | 0.015 |  | 1.00 (refrence) | 1.28 (0.56, 2.90) | 0.561 | 1.99 (0.60, 6.60) | 0.261 |
| Adjusted ^a^ |  |  |  |  |  |  |  |  |  |  |  |
| All-cause | 1.00 (refrence) | 1.67 (0.93, 3.02) | 0.086 | 2.57 (1.51, 4.37) | <0.001 |  | 1.00 (refrence) | 1.32 (1.01, 1.72) | 0.045 | 0.98 (0.54, 1.78) | 0.943 |
| CVD-cause | 1.00 (refrence) | 1.65 (0.64, 4.26) | 0.301 | 1.97 (0.82, 4.70) | 0.128 |  | 1.00 (refrence) | 1.32 (0.88, 1.98) | 0.180 | 0.77 (0.32, 1.85) | 0.566 |
| Cancer-cause | 1.00 (refrence) | 1.07 (0.40, 2.82) | 0.895 | 0.68 (0.15, 3.10) | 0.621 |  | 1.00 (refrence) | 0.98 (0.50, 1.92) | 0.954 | 1.70 (0.42, 6.85) | 0.456 |
| Diabetes-cause | 1.00 (refrence) | 1.13 (0.14, 8.90) | 0.904 | 13.40 (2.10, 65.64) | 0.006 |  | 1.00 (refrence) | 1.20 (0.51, 2.80) | 0.671 | 1.99 (0.46, 8.60) | 0.356 |
| NHANES, National Health and Nutrition Examination Survey; HR, hazard ratio; CI, confidence interval; n, the number; CVD, cardiovascular disease.  ^a^ Model: data were adjusted for adjusted for age, sex, race, education, body mass index, the duration of diabetes, hypertension, smoking, drinking, physical activity, diabetes treatment, complications (retinopathy, CVD, cancer) , ALT, AST, BUN, SUA, TG, TC, HDL, LDL, CRP. | | | | | | | | | | | |
